# The “coracoid tunnel view”: a simulation study for finding the optimal screw trajectory in coracoid base fracture fixation

**DOI:** 10.1007/s00276-019-02274-z

**Published:** 2019-07-04

**Authors:** C. H. van Trikt, J. G. G. Dobbe, J. C. E. Donders, G. J. Streekstra, P. Kloen

**Affiliations:** 1 Department of Orthopaedic Trauma Surgery, Amsterdam UMC, University of Amsterdam, Amsterdam Movement Sciences, Meibergdreef 9, 1105 AZ Amsterdam, The Netherlands; 2 Department of Biomedical Engineering and Physics, Amsterdam UMC, University of Amsterdam, Amsterdam Movement Sciences, Amsterdam, The Netherlands

**Keywords:** Scapula, Coracoid, Fracture, Screw fixation, Fluoroscopy imaging, Radiographic

## Abstract

**Purpose:**

Coracoid fractures represent approximately 3–13% of all scapular fractures. Open reduction and internal fixation can be indicated for a coracoid base fracture. This procedure is challenging due to the nature of visualization of the coracoid with fluoroscopy. The aim of this study was to develop a fluoroscopic imaging protocol, which helps surgeons in finding the optimal insertion point and screw orientation for fixations of coracoid base fractures, and to assess its feasibility in a simulation study.

**Methods:**

A novel imaging protocol was defined for screw fixation of coracoid base fractures under fluoroscopic guidance. The method is based on finding the optimal view for screw insertion perpendicular to the viewing plane. In a fluoroscopy simulation environment, eight orthopaedic surgeons were invited to place a screw down the coracoid stalk through the coracoid base and into the neck of 14 cadaveric scapulae using anatomical landmarks. The surgeons placed screws before and after they received an e-learning of the optimal view. Results of the two sessions were compared and inter-rater reliability was calculated.

**Results:**

Screw placement was correct in 33 out of 56 (58.9%) before, and increased to 50 out of 56 (89.3%) after the coracoid tunnel view was explained to the surgeons, which was a significant improvement (*p* < 0.001).

**Conclusions:**

Our newly developed fluoroscopic view based on simple landmarks is a useful addendum in the orthopaedic surgeon’s tool box to fixate fractures of the coracoid base.

**Electronic supplementary material:**

The online version of this article (10.1007/s00276-019-02274-z) contains supplementary material, which is available to authorized users.

## Introduction

Scapula fractures occur at a rate of 0.7% of all fractures, with an approximate of 3–5% accounted for fractures of the shoulder girdle [5, 6, 17]. It is estimated that fractures of the coracoid process represent 3–13% of all scapular fractures [12]. Fractures of the coracoid are often categorized into two sub-types according to the Ogawa classification, based on their location relative to the coracoclavicular ligament attachment [14]. Type I fractures are located proximal to the coracoclavicular ligament attachment, and Type II fractures distal to the coracoclavicular ligament attachment [14]. Good results are reported by managing Type I fractures with open reduction and internal fixation [1, 8, 13, 14]. However, because of the difficulty in radiographic visualization of the coracoid anatomy [10], placement of a screw down the coracoid pillar through its base into the scapular neck is challenging even for the experienced surgeon. Spatial awareness of the scapula and its surrounding structures is crucial. Misjudgment in drilling angle, position or depth may result in intra-articular, hardware, and/or neurovascular damage [8, 11, 21]. During surgery, fluoroscopy is used as guidance for screw fixation of a Type I coracoid fracture. Although fluoroscopy in anterior–posterior view, axillary scapular view and lateral scapular view may be useful in guiding the surgeon, it does not render the desired geographical insight due to the scapula’s complex propeller blade-like shape.

Previous studies attempted to increase anatomical insight of the coracoid by analyzing the morphometric data [4, 7, 16]. Another study developed a new radiographic technique to visualize the individual pillars of the coracoid by standardizing the standing position of the patient and the radiographic beam angulation [2]. However, these reported morphometric data of the coracoid might not be applicable to every patient undergoing surgery as there was substantial heterogeneity regarding the way of measurement. In addition, the developed radiographic techniques might be susceptible to imprecise imaging depending on the position of the patient on the operating table. Therefore, a new radiographic approach solely based on anatomical landmarks (i.e., without considering the morphometric data of the coracoid as guidance) might be more practical and universally applicable.

The primary aim of this study is to establish a new fluoroscopic view based on anatomical landmarks that facilitate the surgeon in screw fixation of Type I coracoid fractures. And the secondary aim of this study is to validate the view by evaluating the accuracy and the inter-rater reproducibility of using or not using the configuration of anatomical landmarks of the optimal view for coracoid screw fixation. We hypothesize that this new configuration of landmarks is a useful addendum for the orthopaedic surgeon in fixation of coracoid base fractures.

## Materials and methods

### Fluoroscopy-mimicking and virtual screw placement software

Custom-made software was used to make a virtual 3D representation of each scapula by segmentation from its original CT scan. This application was further used to create reconstructions of virtual bone surface models and digitally reconstructed radiographs out of CT data of each scapula effectively mimicking fluoroscopy imaging [18, 20]. Brightness and contrast of all virtual fluoroscopic images were chosen to reflect actual fluoroscopic imaging. In addition, the application enables the operator to rotate and translate a CT volume freely in 3D space to mimic fluoroscopy imaging of the scapulae at different orientations of the X-ray system.

### Data acquisition

A total of 24 cadaveric scapulae (mean age: 76 years; range: 41–91 years) were scanned individually with a high-resolution CT scanner (Philips Healthcare, Best, The Netherlands) (120 kV, 150 mAs, slice thickness 0.9 mm, slice increment 0.45 mm) of which 10 were used to establish an optimal fluoroscopic view, the coracoid tunnel view (CTV) and protocol for optimal screw placement for coracoid base fracture fixation. The remaining scapulae (*n* = 14) were used to evaluate screw placement with and without providing information about landmark arrangement in the CTV. The cadaveric scapulae were obtained from the Department of Medical Biology, Section Anatomy & Embryology, Academic Medical Center, Amsterdam Movement Sciences, University of Amsterdam, Amsterdam, The Netherlands; and were dissected and macerated.

### Defining the CTV

**Fig. 1:**
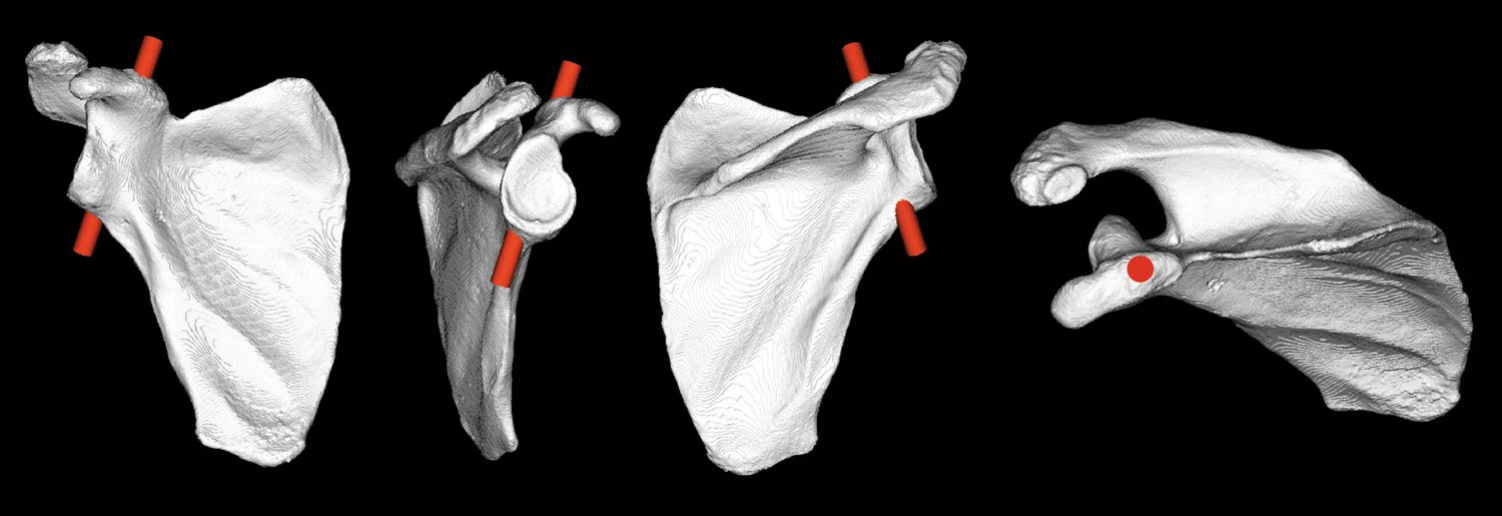
The entry and exit points of an optimally placed virtual screw down the coracoid tunnel. The right-most viewing plane is perpendicular to the screw and is representative for the optimal view in fluoroscopy imaging

The optimal passageway of a screw down the coracoid stalk, through the coracoid base and into the neck of the scapula is described in this study as the coracoid tunnel (Fig. 1). The method for inserting a screw through the coracoid tunnel is based on finding the optimal fluoroscopic view (CTV) that is perpendicular to the coracoid tunnel, i.e., with the central axis of the coracoid tunnel in line with the central X-ray beam. In this view, the optimal screw orientation is achieved if the screw is visualized as a dot in the fluoroscopic view.

**Fig. 2:**
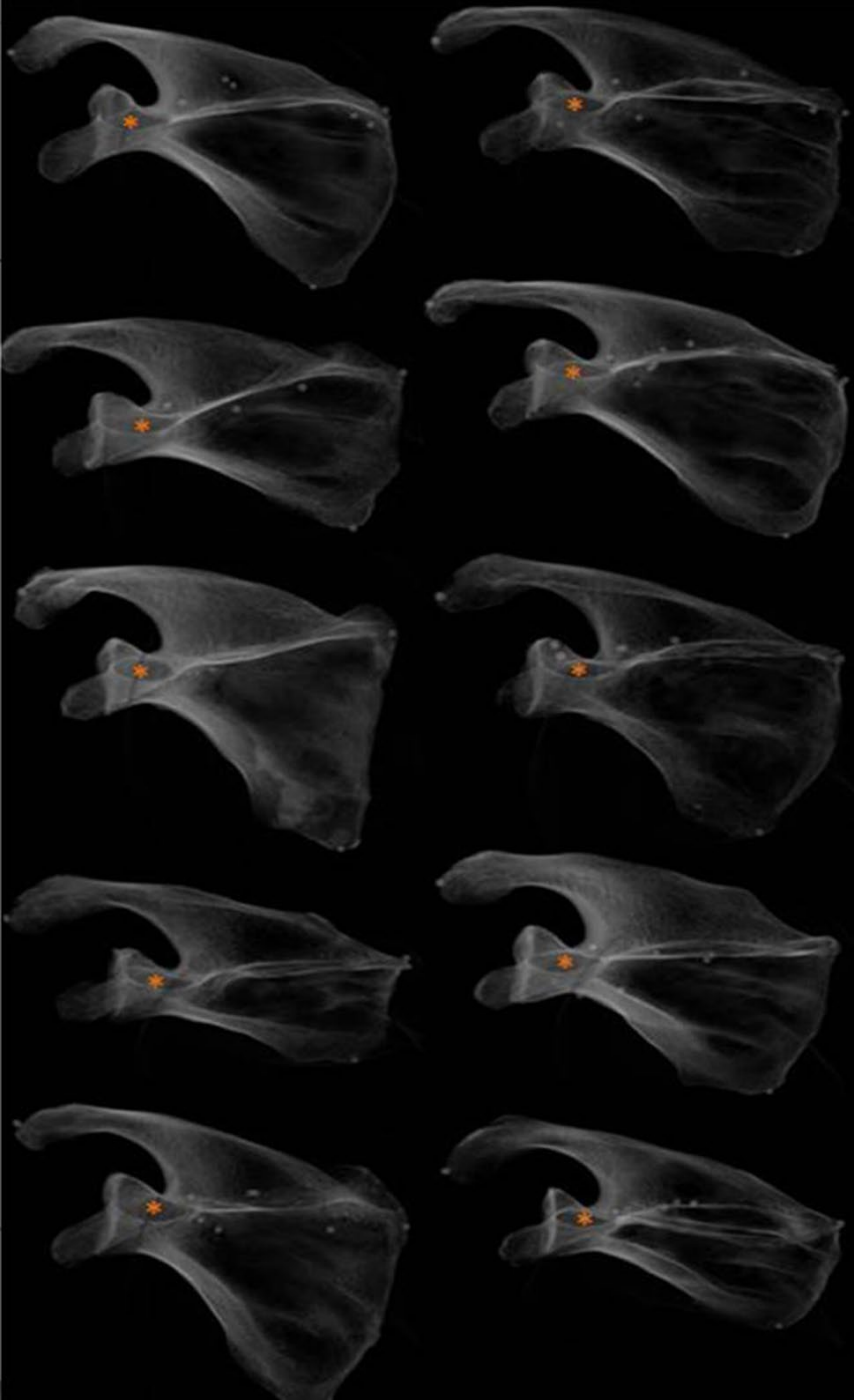
The similarity of the oval shape of the coracoid process base is well shown in the coracoid optimal view for 10 different scapulae. *Indicates the virtual screw insertion point

Ten virtual bone surface models of the scapula were analyzed to determine the entry and exit points of an optimally placed screw down the coracoid stalk, through the coracoid base and into the neck of the scapula. To this end, we placed a virtual cylinder in each segmented scapula model, representing a fixation screw, as precise as possible in the middle of the cross-sectional area of the coracoid base and subsequently adjusted the diameter, so that it does not bulge outside the coracoid tunnel (Fig. 1). These cylinders were oriented perpendicular to the plane of the optimal fluoroscopic view, i.e., CTV (Fig. 2). A correctly placed virtual screw down the coracoid tunnel was visible in the CTV as a dot. The shape of the coracoid tunnel in the CTVs was similar for all 10 scapulae (Fig. 2).

**Fig. 3:**
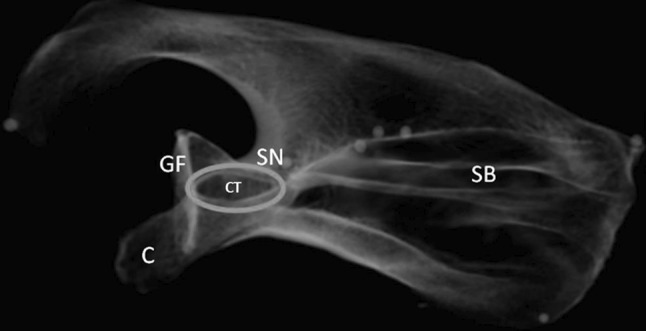
The optimal view of the coracoid tunnel (CT) is based on four anatomical landmarks: coracoid (C), glenoid fossa (GF), scapular notch (SN) and superior scapular border (SB)

We then identified an adequate arrangement of radiographic landmarks that can be used to reproduce the CTV. This so-called CTV is based on relative positions of four anatomical landmarks (Fig. 3): coracoid, glenoid fossa, scapular notch and superior scapular border.

To obtain the CTV for placement of a coracoid base fixation screw, we used these landmarks to yield the following protocol.

**Fig. 4:**
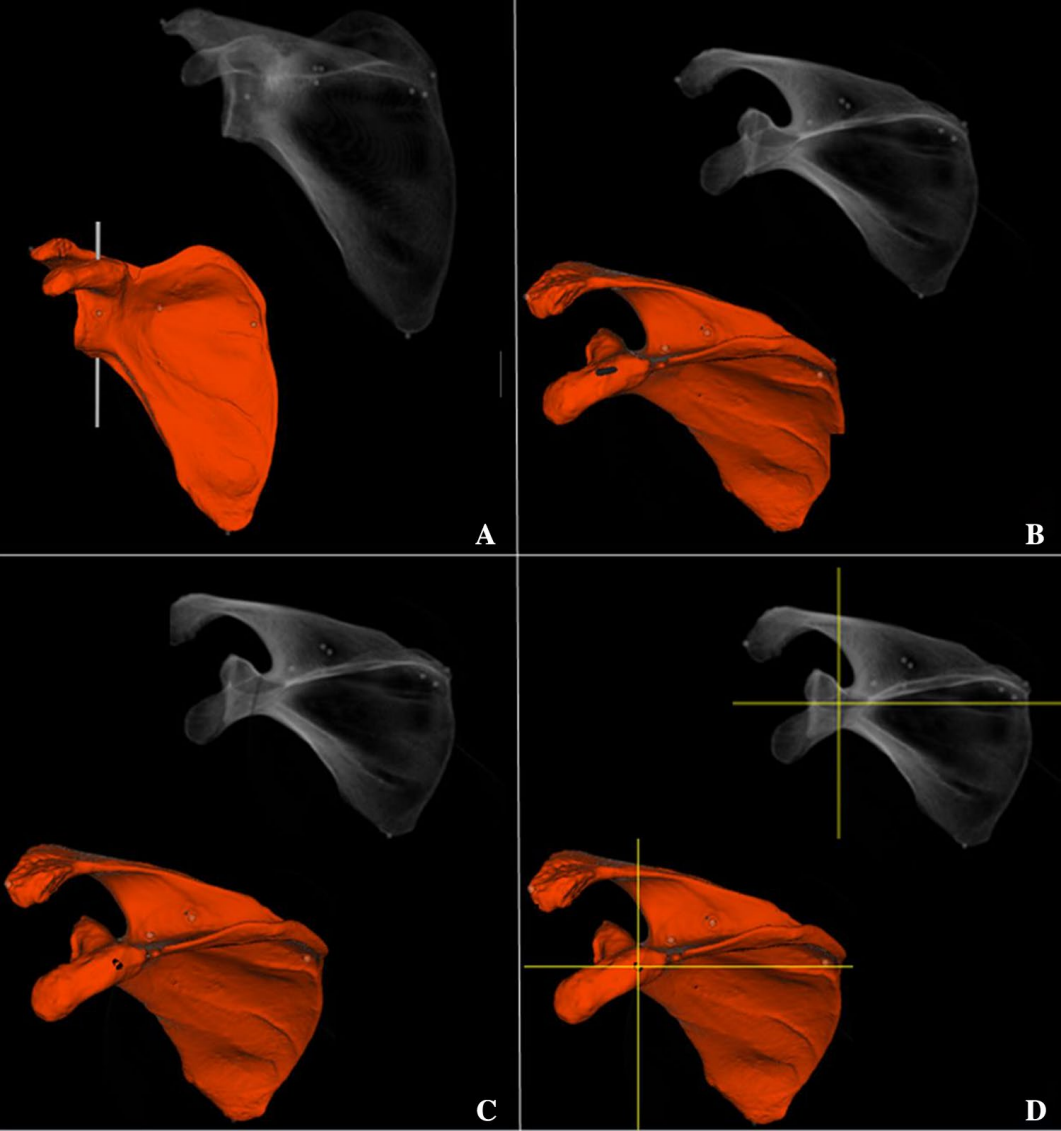
CTV protocol for fluoroscopy imaging: **a** view scapula in radiographic anterior–posterior view: identify the glenoid fossa, coracoid, acromion, scapular notch, superior scapular border, medial border, inferior border and scapula spine; **b** align viewing angle in cephalad direction until oval shaped tunnel appears; **c** adjust viewing angle until glenoid fossa is parallel to drilling direction; **d** the crosshair marks the optimal screw position

CTV identification protocol (Fig. 4 and supplementary video: “coracoid tunnel view”):

1. View scapula in radiographic anterior–posterior view: identify the glenoid fossa, coracoid, acromion, scapular notch, superior scapular border, medial border, inferior border and scapula spine.
2. Change viewing angle in cephalad direction until oval shaped tunnel (= coracoid tunnel) is projected between coracoid tip, glenoid fossa, scapular notch and superior scapular border.
3. Adjust viewing angle until glenoid fossa is parallel to drilling direction. Make sure that superior scapular border is kept superimposed into roughly one line.

### Screw placement evaluation before/after CTV instructions

Fourteen virtual fluoroscopic scapula images were used to evaluate the protrusion of cylinders representing virtually placed fixation screws, down the coracoid tunnel using the simulated fluoroscopy images of the CTV. In this study, we consider a screw correctly placed down the coracoid tunnel if the following criteria are met:

1. The screw must be placed down the coracoid stalk, through the coracoid base and into the neck of the scapula.
2. The screw must be placed parallel to the glenoid fossa.
3. The screw must be placed without violating or penetrating the bony borders of the coracoid tunnel.

Eight orthopaedic surgeons were invited for two sessions to determine the optimal fluoroscopic view for placement of virtual screws down the coracoid tunnel. Seven of these scans were used to evaluate screw placement before explaining the CTV (before CTV instruction), the remaining seven were used to evaluate screw placement after giving CTV instructions (after CTV instruction). We provided the surgeons prior to the experiment with an instructional course and trial run on software handling. In the “before CTV instruction” session, we instructed the surgeons to place a virtual screw down the coracoid tunnel in seven individual scapulae using whichever viewing angle, scapula position and anatomical landmarks they preferred as long as they complied with our predefined criteria. The viewing angle in the fluoroscopy mimicking system was equivalent to the drilling angle of the virtual screws. The exact drilling location was marked with a crosshair on the screen. Once the surgeon was satisfied with the crosshair location, the cylinder representing a virtual screw was placed perpendicular to the viewing plane down the coracoid tunnel.

In the “after CTV instruction” session, we handed the surgeons the protocol to identify the CTV. They were instructed on how to use the anatomical landmarks of the scapula to obtain the CTV. Instructions given to each surgeon were standardized and consisted of four comprehensive steps, explanatory figures and a scapula bone model for assisted spatial orientation (Fig. 4). After the instructions, the surgeons proceeded with the experiment. They once again had to eject a virtual screw as explained above but now strictly using our developed CTV protocol.

The screws placed by each surgeon in both sessions were evaluated for coracoid tunnel protrusion in accordance with our predefined criteria by an experienced orthopaedic surgeon (PK) who was blinded for the applicable session. The length and depth of the placed virtual screws were not taken into account in the analysis. During the experiment, 112 virtual screws were placed (8 surgeons placed in each of the 14 individual scapulae one virtual screw) down the coracoid tunnel. All virtual screws were rated following a “Correct” or “Incorrect” binary scoring method (Correct = “virtual screw” in compliance with the above-mentioned criteria, Incorrect = “virtual screw” not in compliance with the above-mentioned criteria).

### Statistical analysis

The results of the before and after CTV instruction sessions were collected and presented in a 2 × 2 cross table. Subsequently, a McNemar test for paired proportions data was performed to establish if screw placement with the CTV protocol was significantly better.

Intraclass correlation coefficient (ICC) estimates and their 95% confident intervals (CI) were calculated based on an average measurement, absolute agreement, two-way mixed model. ICC values less than 0.5 are indicative of poor reliability, values between 0.5 and 0.75 indicate moderate reliability, values between 0.75 and 0.9 indicate good reliability, and values greater than 0.9 indicate excellent reliability [15].

Post hoc power analysis using a sample size of 56 and alpha level of *p* < 0.05 revealed a statistical power of 0.94. The statistical analyses were calculated using SPSS statistical package version 24 (SPSS Inc., Chicago, IL, USA).

## Results

### Screw placement evaluation with/without CTV instruction

**Fig. 5:**
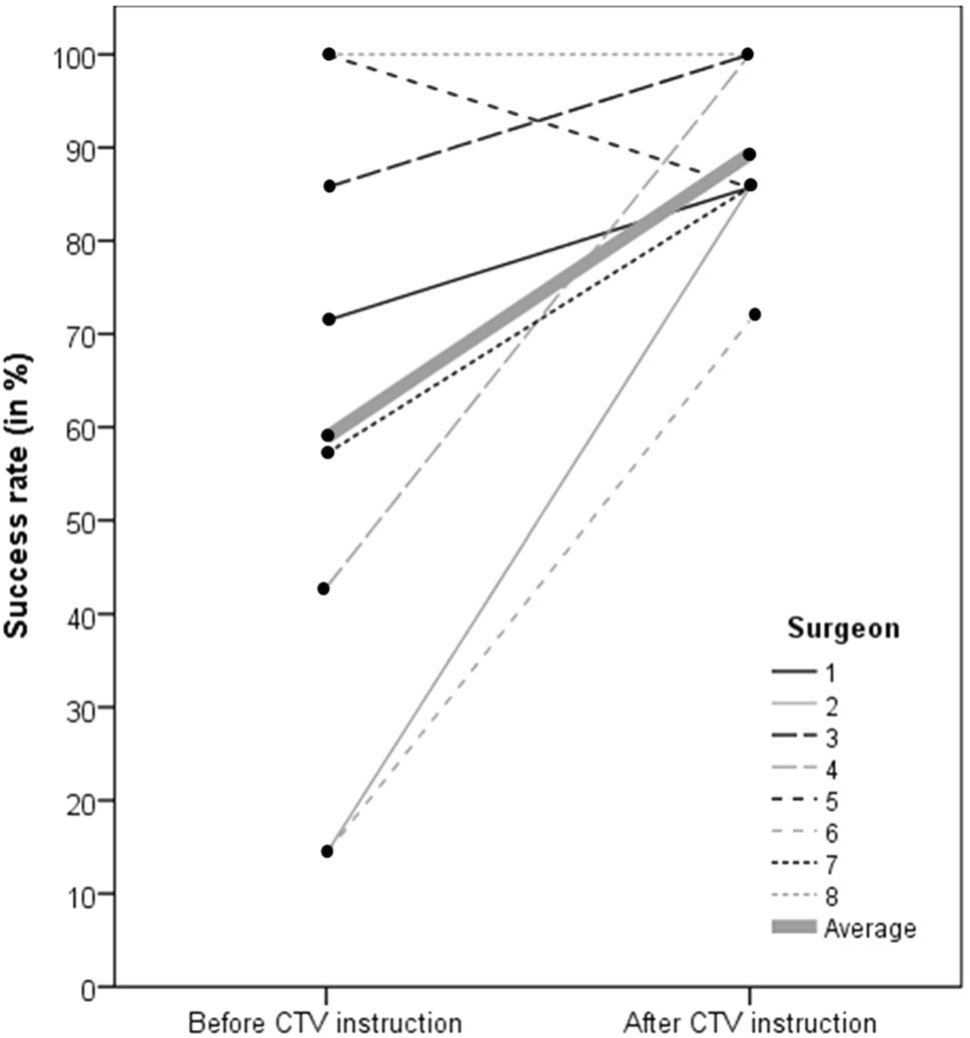
Success rate for placing virtual screws down the coracoid tunnel using fluoroscopy imaging. Each line connects the success rate of a surgeon before and after CTV instructions

In the “after CTV instruction” session, virtual screw placement was correct in 50 out of 56 (89.3%) of the cases, whereas this was only 33 out of 56 (58.9%) in the “before CTV instruction” session. Six of eight surgeons showed an overall improvement in correct placement of virtual screws down the coracoid tunnel after receiving the instructions. See Fig. 5 for evaluation in percentages of correctly placed virtual screws down the coracoid tunnel.

The McNemar test confirmed that the correct virtual screw placement improvement by 30.4% from the “before CTV instruction” session to the “after CTV instruction” session was statistically significant (*p* < 0.001).

A moderate degree of reliability was found between the two sessions. The average was 0.675 with a 95% CI from 0.382 to 0.879 (*F* (13,78) = 3.077, *p* < 0.001).

## Discussion

In the current study, we presented a protocol for fixation of coracoid base fractures using a newly developed fluoroscopic view: the CTV. We determined four radiographic landmarks to easily replicate this view: the coracoid, glenoid fossa, scapular notch and superior scapular border. The proposed imaging protocol increased the rate of correctly placed virtual screws according to our predefined criteria from 59% in the “before CTV instruction” session to 89% in the “after CTV instruction” session. Moreover, six out of eight surgeons improved their screw placement skills after making use of the CTV. One of these two surgeons had a perfect score in both sessions; therefore, an improvement was not observed.

During the development of the CTV imaging protocol, we noticed that all coracoid tunnels were similar, although, unique for each individual scapula. We, therefore, chose to standardize the drilling angle in our predefined criteria by following the surgical strategy of Ogawa and colleagues, which is drilling parallel to the glenoid fossa, for the reduction and stabilization of the coracoid base fracture [13]. More importantly, by drilling parallel to the glenoid fossa, the danger zone (topographic distribution of suprascapular nerve and the ascending part of the circumflex scapular artery) as described by Wijdicks and colleagues is most likely avoided, consequently reducing neurovascular risks [21].

Several studies analyzed the morphometry of the coracoid process to determine ideal internal screw fixation of the coracoid [4, 9, 19]. However, during an intra-operative intervention, the surgeon relies primarily on fluoroscopy to correctly place a screw. For this last step, we designed and investigated a useful method in addition to this work.

Few articles address the surgical treatment of coracoid fractures specifically. Bhatia and colleagues gave a detailed account on fixating coracoid fractures percutaneously using two clearly defined fluoroscopic views as a guide [2]. Ogawa and colleagues described an open surgical technique where the direction of the Kirschner wire is placed under the guidance of fluoroscopic images, and the position of the Kirschner wire was confirmed by tactual exploration with the fingertip at the coracoid base and penetrating sensations of the drill [13]. In a case report by Kawasaki and colleagues, a technique was described for reducing coracoid base fractures using only a fluoroscopic view in the axial plane of the coracoid base [9]. Their finding of tilting the C-arm until an axial circle of the coracoid base is visible is somewhat in agreement with the results of our study and support the idea of a CTV; which is, the oval-shaped structure of the coracoid base is only visible in a certain fluoroscopic viewing angle.

**Fig. 6:**
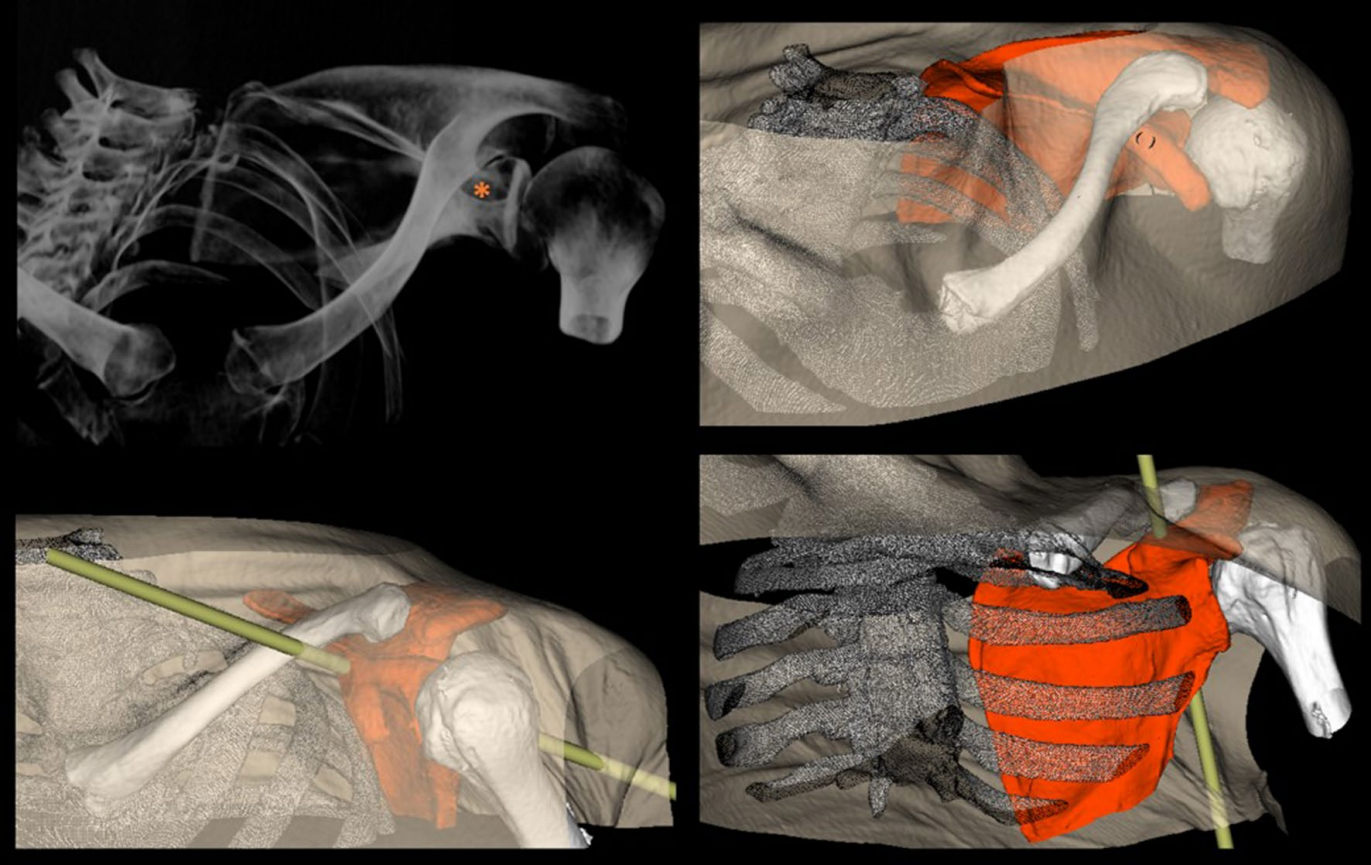
A virtual representation of clinical setting with real projection of surrounding bones. The coracoid tunnel (“*”) is visible only in correct alignment of the anatomical landmarks

The simulation design of our study prevents unnecessary radiation exposure to patients and surgeons. However, our experimental study may lead to an underestimation of the difficulty in reproducing the CTV in an actual intra-operative setting. The use of macerated scapulae was very helpful in defining the optimal CTV; however, the CTV in patients will show over-projection of surrounding bones, which may render finding the CTV more difficult in patients than in our study [2, 3, 9, 13]. Finally, the virtual procedures were only performed in intact scapulae. Thus, the surgeons were placing virtual screws in perfect samples. In spite of these limitations, we believe that the presented protocol can be of aid to the surgeon, because only in case of correct alignment of the anatomical landmarks (i.e., coracoid tip, glenoid fossa, scapular notch and superior border), the coracoid tunnel is visible and correct coracoid base fixation is possible (Fig. 6).

## Conclusions

In conclusion, our study demonstrates that the CTV is a useful radiographic technique for the orthopaedic surgeon to fixate fractures of the coracoid base. The anatomical landmarks are comprehensive and easy to find. In addition, it showed an improvement in correct (virtual) placement of screws according to our predefined criteria. Future clinical studies should validate the protocol and also determine the accuracy of the CTV in a clinical setting.

## Electronic supplementary material

Below is the link to the electronic supplementary material.

Supplementary material 1 (MP4 25604 kb)

## References

[1] Anavian J, Wijdicks CA, Schroder LK, Vang S, Cole PA (2009). Surgery for scapula process fractures: good outcome in 26 patients. Acta Orthop.

[2] Bhatia DN (2012). Orthogonal biplanar fluoroscopy-guided percutaneous fixation of a coracoid base fracture associated with acromioclavicular joint dislocation. Tech Hand Up Extrem Surg.

[3] Bhatia DN, Dasgupta B, Rao N (2013). Orthogonal radiographic technique for radiographic visualization of coracoid process fractures and pericoracoid fracture extensions. J Orthop Trauma.

[4] Bhatia DN, de Beer JF, du Toit DF (2007). Coracoid process anatomy: implications in radiographic imaging and surgery. Clin Anat.

[5] Butters KP, Rockwood CA (1990). The scapula. The shoulder.

[6] Court-Brown CM, Wood AM, Aitken SJI (2008). The epidemiology of acute sports-related fractures in adults. Injury.

[7] Fathi M, Cheah PS, Ahmad U, Nasir MN, San AA, Abdul Rahim E, Hussin P, Mahmud R, Othman F (2017). Anatomic variation in morphometry of human coracoid process among Asian population. Biomed Res Int.

[8] Hill BW, Jacobson AR, Anavian J, Cole PA (2014). Surgical management of coracoid fractures: technical tricks and clinical experience. J Orthop Trauma.

[9] Kawasaki Y, Hirano T, Miyatake K, Fujii K, Takeda Y (2014). Safety screw fixation technique in a case of coracoid base fracture with acromioclavicular dislocation and coracoid base cross-sectional size data from a computed axial tomography study. Arch Orthop Trauma Surg.

[10] Kim KC, Rhee KJ, Shin HD, Kim DK, Shin HS (2009). Displaced fracture of the coracoid process associated with acromioclavicular dislocation: a two-bird-one-stone solution. J Trauma.

[11] Lo IK, Burkhart SS, Parten PM (2004). Surgery about the coracoid: neurovascular structures at risk. Arthroscopy.

[12] McGinnis M, Denton JR (1989). Fractures of the scapula: a retrospective study of 40 fractured scapulae. J Trauma.

[13] Ogawa K, Matsumura N, Ikegami H (2012). Coracoid fractures: therapeutic strategy and surgical outcomes. J Trauma Acute Care Surg.

[14] Ogawa K, Yoshida A, Takahashi M, Ui M (1997). Fractures of the coracoid process. J Bone Joint Surg Br.

[15] Portney LG, Watkins MP (2009). Foundations of clinical research: applications to practice.

[16] Rios CG, Arciero RA, Mazzocca AD (2007). Anatomy of the clavicle and coracoid process for reconstruction of the coracoclavicular ligaments. Am J Sports Med.

[17] Rowe CR (1963). Fractures of the scapula. Surg Clinics of North Am.

[18] Schroeder W, Martin K, Lorensen B (2006). The visualization toolkit: an object-oriented approach to 3D graphics.

[19] von Schroeder HP, Kuiper SD, Botte MJ (2001). Osseous anatomy of the scapula. Clin Orthop Relat Res.

[20] Wiggers JK, Snijders RM, Dobbe JGG, Streekstra GJ, den Hartog D, Schep NWL (2017). Accuracy in identifying the elbow rotation axis on simulated fluoroscopic images using a new anatomical landmark. Strateg Trauma Limb Reconstr.

[21] Wijdicks CA, Armitage BM, Anavian J, Schroder LK, Cole PA (2009). Vulnerable neurovasculature with a posterior approach to the scapula. Clin Orthop Relat Res.

